# Individual identification of endangered amphibians using deep learning and smartphone images: case study of the Japanese giant salamander (*Andrias japonicus*)

**DOI:** 10.1038/s41598-023-40814-1

**Published:** 2023-09-27

**Authors:** Kosuke Takaya, Yuki Taguchi, Takeshi Ise

**Affiliations:** 1 Graduate School of Agriculture, Kyoto University, Kyoto, Japan; 2 Hiroshima City Asa Zoological Park, Hiroshima, Japan; 3 Field Science Education and Research Center, Kyoto University, Kyoto, Japan

**Keywords:** Ecology, Zoology

## Abstract

Information obtained via individual identification is invaluable for ecology and conservation. Physical tags, such as PIT tags and GPS, have been used for individual identification; however, these methods could impact on animal behavior and survival rates, and the tags may become lost. Although non-invasive methods that do not affect the target species (such as manual photoidentification) are available, these techniques utilize stripes and spots that are unique to the individual, which requires training, and applying them to large datasets is challenging. Many studies that have applied deep learning for identification have focused on species-level identification, but few have addressed individual-level identification. In this study, we developed an image-based identification method based on deep learning that uses the head spot pattern of the Japanese giant salamander (*Andrias japonicus*), an endemic and endangered species in Japan. We trained and evaluated a dataset collected over two days from 11 individuals in captivity, which included 7075 images taken by a smartphone camera. Individuals were photographed three times a day at approximately 11:00 (morning), 15:00 (afternoon), and 18:00 (evening). As a result, individual identification by our method, which used the EfficientNetV2 achieved 99.86% accuracy, kappa coefficient of 0.99, and an F1 score of 0.99. Performance was lower for the evening  model than for the morning and afternoon models, which were trained and evaluated using photographs taken at the corresponding time of the day. The proposed method does not require direct contact with the target species, and the effect on the animals is minimal; moreover, individual-level information can be obtained under natural conditions. In the future, smartphone images can be applied to citizen science surveys and individual-level big data collection, which is difficult using current methods.

## Introduction

Individual identification of wildlife provides fundamental information for ecological studies and conservation efforts^1,2^. For example, individual-based studies combined with mark-recapture methods offer estimates of population size, survival and reproduction rates, and immigration and emigration rates. In addition, health status indicators, such as weight and presence of parasites, and behavior variations are important in behavioral evolution and urban adaptation and can be revealed through individual identification. These findings can contribute to answering ecological and evolutionary questions and facilitating the conservation of endangered species^3^.

Individual identification is essential for collecting information at the individual level. There are two main types of approaches: invasive and non-invasive. Invasive methods include blood and tissue sampling and the attachment of physical tags, GPS, and radio transmitters. However, it can be difficult to capture target species in the field, and concerns have been raised about the negative effects of tag attachment^4–7^. Additionally, research permits are required for certain animals, and obtaining such permits is particularly difficult for endangered species. Furthermore, tags often have a short lifetime and are sometimes lost^8^, which represents a challenge when applied to long-lived organisms. Although these issues can be addressed by non-invasive methods, such as genetic analysis using DNA in feces and hairs, collecting samples from aquatic organisms, such as amphibians, is not easy because feces diffuse in the water. In addition, DNA analysis is expensive and requires fresh samples.

Biometric identification techniques, such as manual photoidentification, are inexpensive and can identify individuals without harming animals. These methods have several advantages, such as no animal capture, no loss of tags and no effect on animal behavior, because individual-specific patterns, such as stripes and spots, can be utilized for individual identification, which is beneficial for the study of endangered species including individual identification of cetaceans^9^, sea lions^10^, lions^11^, polar bears^12^, African elephants^13^, and sea turtles^8,14^. Such methods are alternatives to invasive methods, although they cannot be applied when natural markings are absent^15^. However, as the number of individuals increases, image classification requires more time and effort; because it is difficult to process large datasets.

In recent years, computer vision has attracted attention as a method of overcoming the challenges of manual photoidentification. Deep learning, such as convolutional neural networks (CNNs), is a new approach for automatically extracting features from large amounts of data. Its implementation in various fields is rapidly advancing with improvements in computing power, such as graphics processing units (GPUs). This technique, which has been used for human facial recognition, was first applied to animal identification in 2014^16^. The target species for individual identification have mainly been mammals^17–20^, although the method has also been applied to birds and reptiles, with large research bias observed according to the taxonomic group^8,21^. Pattern recognition has been used to identify amphibian individuals^22^, but image recognition based on deep learning has not yet been applied. Amphibian populations are declining globally, with 41% of amphibians listed as threatened by extinction on the IUCN Red List^23^. Therefore, the application of deep learning is needed for this taxon which is a high conservation priority^24,25^.

The Japanese giant salamander (*Andrias japonicus*) is one of the world’s largest amphibians and endemic species and reaches 150 cm in total length, and it is distributed in the up-streams and middle rivers of western Japan^26^. This species has primitive morphological features similar to those of fossil species and a life span of over 60 years. Their diet is carnivorous, including fish and crabs, and they are top predators in the stream ecosystem. Although this species is protected by law, its population has declined because of habitat modification and fragmentation^27^. Therefore, it has been listed as a vulnerable species by the IUCN and Ministry of the Environment’s Red List. Furthermore, hybridization of this species with the Chinese giant salamander (*Andrias cf. davidianus*) has become a problem that requires immediate conservation efforts. Currently, PIT tags are mainly used to identify individuals of this species; however, capture is necessary to insert tags. Before PIT tags became popular, spot patterns on their bodies were mainly used for identification by experts capable of identifying individuals from their unique patterns.

In this study, we aimed to identify individuals of *A. japonicus* via deep learning using spot patterns captured by smartphone. Individual identification from images is a non-contact, non-destructive, and low-cost method that can be applied to other species. Because conservation practices are expensive, inexpensive identification methods will enable conservation measures to be applied for more species. In particular, the conservation of charismatic species, mainly mammals, has generally captured public interest and been the focus of efforts. However, less charismatic animals, such as amphibians, have not received sufficient financial support for their conservation^28,29^. Therefore, inexpensive identification can significantly contribute to the conservation of amphibians.

## Materials and methods

### Ethics declarations

*A. japonicus* are protected by the Law for the Protection of Cultural Properties as a “National Natural Monument”. Therefore, this study was approved by the Hiroshima City Asa Zoological Park, which has permission from the Agency for Cultural Affairs and is categorized as a non-invasive study (Permission number: 4 Buncho No. 5012).

### Natural markings

The markers used for individual identification should be permanent, distinctive of the animals, and universally displayed throughout the population, and they should also be measurable using a recording device^30^. In addition, a suitable measurement region for the target species must be determined. *A. japonicus* has spot patterns all over its body, and the head and tail spots have been used to identify individuals^31^. A previous study showed that about eight years of continuous identification could be conducted by their body pattern^32^. Therefore, we selected the head as the measurement region because spots were clearly observed and easily photographed by the camera.

### Image acquisition

In this study, 11 individuals kept at the Conservation breeding facility of *A. japonicus* in Hiroshima City Asa Zoological Park were used (Supplementary Fig. 1). This facility has been used for researching and breeding *A. japonicus* successfully in captivity since 1971. A smartphone (iPhone11 equipped with a 12-megapixel camera) was used for image acquisition. Obtaining optimal images on land was difficult because of the body surface reflection and active movement of the individuals. Therefore, we photographed *A. japonicus* in water using a camera above the water. The head spot of *A. japonicus* was recorded at approximately 60 cm from the camera, and the water depth was about 20 cm. In our experiment, 60 cm was the appropriate distance because they were about 60–90 cm in total length, and the head spot could be clearly photographed. Although we photographed individuals in the zoological facility, the optimal distance could differ depending on the size of the individual and the water clarity, especially in the field. Because reflections on the water surface were a problem with this method, the photographer held a black umbrella to suppress reflections on the water surface and conducted shooting under the umbrella (Supplementary Fig. 2). This method was effective because we photographed in an environment without water flow. The water’s surface will be more complex in the natural environment, but blocking direct light on the surface with an umbrella would work adequately. In severe water surface reflection, deflection (polarization) filters could also be effective. For example, the effectiveness of the polarization filter has already been confirmed for salmon surveys in rivers^33^. Spots were photographed on video and converted to images at ten frames per second using the Free Video to JPG Converter software program, and these images were used as training and test images. In this study, we photographed images three times a day to investigate the effect of light conditions on model accuracy. Photographing was performed on August 20–21, 2022. Each video recording lasted approximately 30 s, and shooting was conducted three times a day at approximately 11:00, 15:00, and 18:00, which are defined as morning, afternoon, and evening, respectively. The solar radiation measured at the Hiroshima Local Meteorological Observatory during each hour on August 20 was 2.00, 0.78, and 0.23 (MJ/m^2^). Similarly, August 21 was 0.98, 2.72, and 0.70 (MJ/m^2^). Although testing at night light conditions is important because *A. japonicus* are nocturnal, our study aimed to verify the feasibility of individual identification based on the spot patterns. Therefore, we used images during the daytime because photographing is relatively easy. 

**Figure 1:**
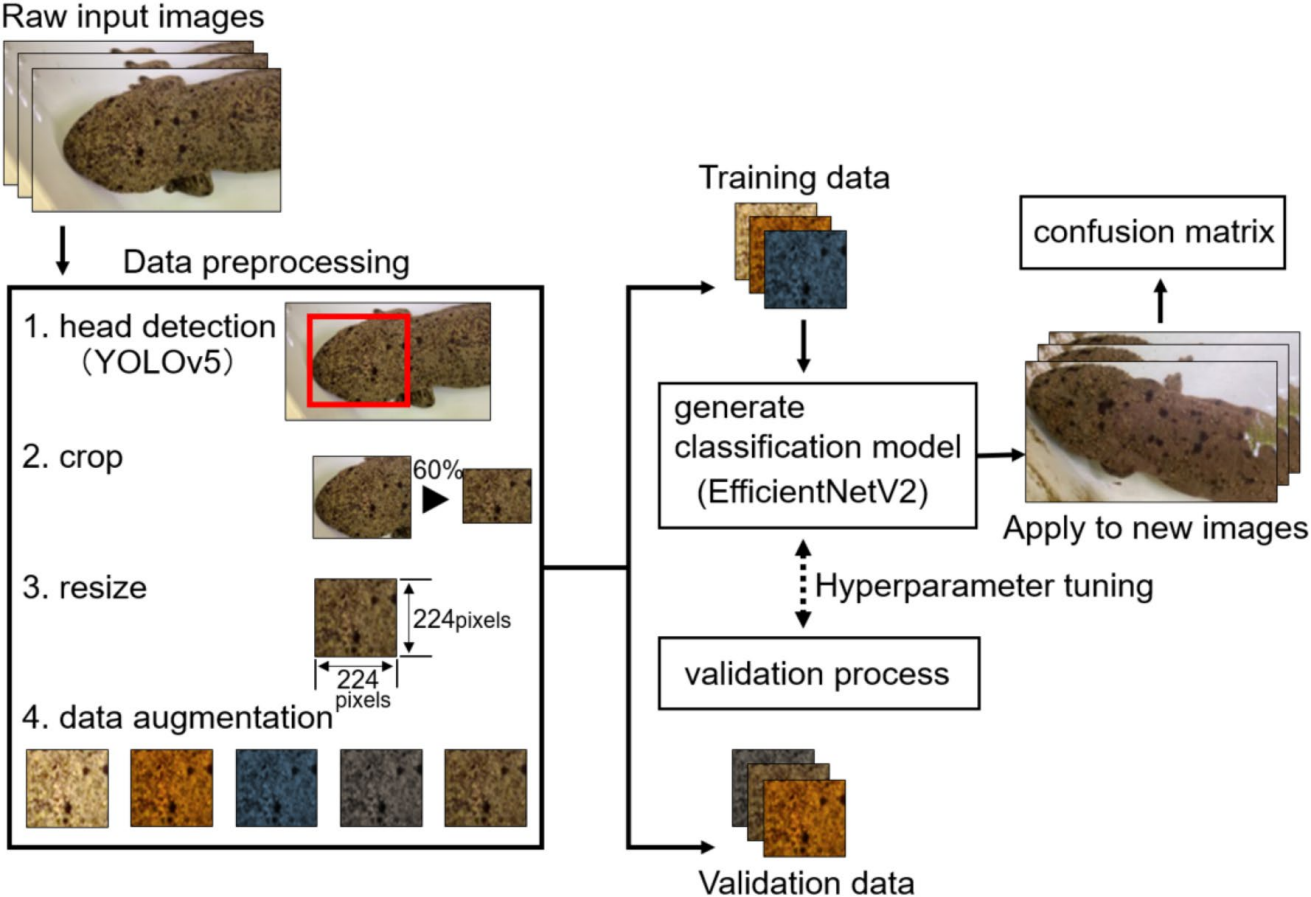
Framework of the classification model for identifying *A. japonicus* using smartphone photographs.

**Figure 2:**
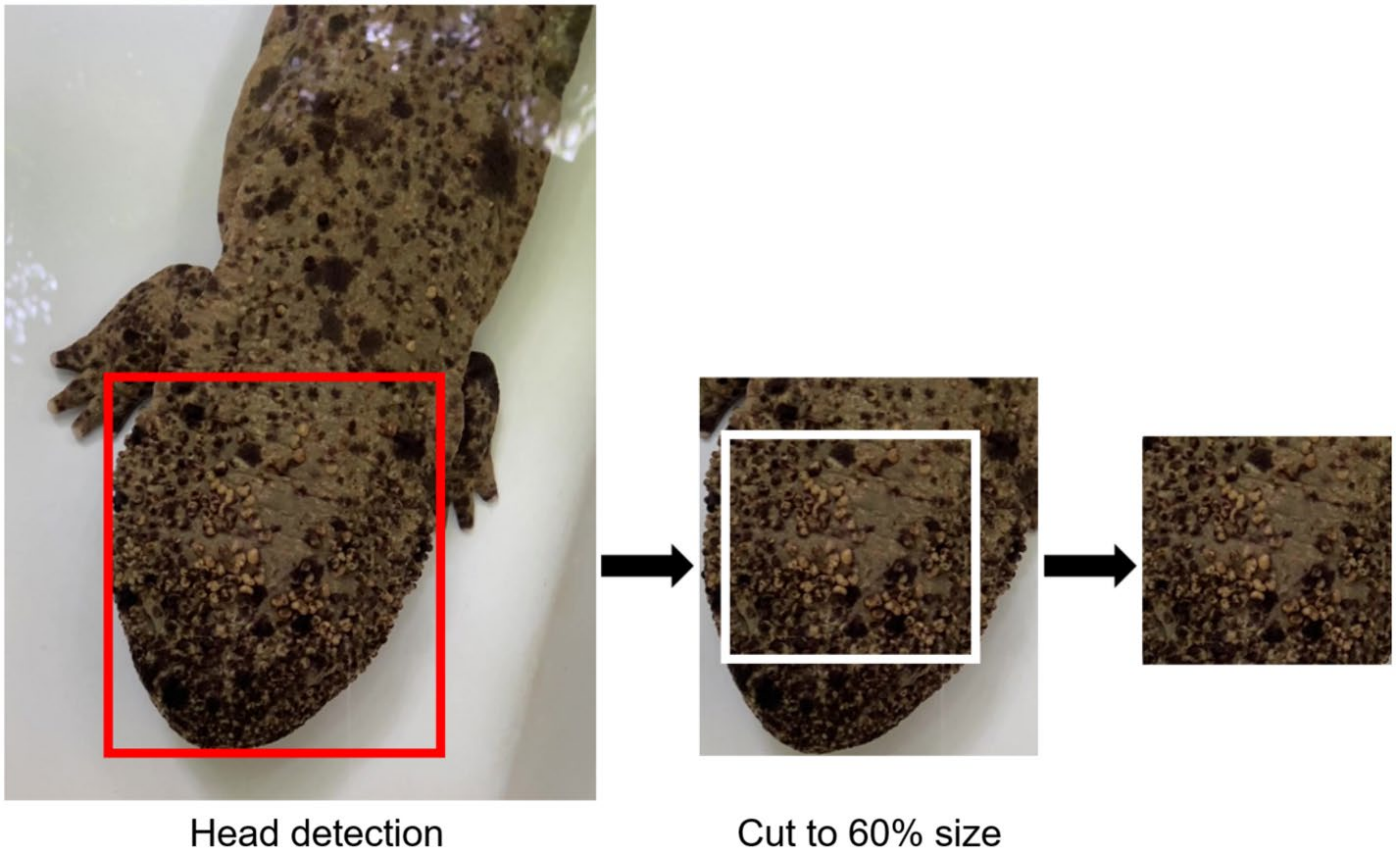
The head was detected by Yolov5 (red box); the image also includes the background. Cropping the head image to 60% of its original size (white box) produced an image that only included spots.

The framework employed in this study is illustrated in Fig. 1. The head in the image was automatically detected using YOLOv5^34^. Annotation data were created using the LabelImg annotation tool^35^. First, an image of the *A. japonicus* was loaded using this software (Supplementary Fig. 3). In this case, 10 images per individual were selected from 11 individuals and used for the annotation data. Next, a rectangle was created to include only the head. Finally, the rectangles were labeled “head” and the output format was the YOLOv5 format to train the model. The detection model was tested against all of 11 individuals to quantify the performance. After detecting the head in this model, a rectangle in a similar shape to the detected region was created in the center of the region. The size of a similar rectangle was set to 60% area of the detected region and cropped automatically (Fig. 2; Table 1). Although the head image detected by YOLOv5 may contain a background, only an image of the spot patterns were created by cropping at 60%. The training and test images were resized to 224 × 224 pixels because the size varied from image to image. This study used the images obtained on August 20 for training and August 21 for testing (Supplementary Table 1). We augmented training datasets and randomly divided those images into training (70%) and validation (30%). Details about augmentation are described in the next section.

**Table 1:** Dataset summary.

Individual	Traning (August 20)			Test (August 21)		
	Morning	Afternoon	Evening	Morning	Afternoon	Evening
1	112	110	98	107	87	106
2	109	105	121	106	100	103
3	87	100	101	102	86	108
4	94	110	115	105	82	91
5	97	124	104	103	105	103
6	112	104	104	108	105	100
7	107	108	98	107	106	101
8	77	158	127	103	88	187
9	102	108	98	105	135	101
10	103	160	106	103	104	101
11	109	122	103	104	83	147

Date, time, and purpose of the images in each individual.

### Image augmentation

Augmentation is performed to prevent overfitting. The orientation of the individual in the image differs because it moves during the shooting; therefore, rotation and crop processing were added to identify, regardless of the direction of the measurement region. In addition, brightness, Gaussian noise, color jitter, and saturation processing were performed because the light conditions in the image were not uniform. The augmentation process was applied to each parameter with a probability of 50%. For example, applying rotation and cropping will result in the following three patterns of images: (1) both processes are applied, (2) either process is applied or not, and (3) neither process is applied. 

### EfficientNetV2

In this study, EfficientNetV2, an improved version of EfficientNet, was used for classification. EfficientNet is a convolutional neural network model that achieves more efficient performance by uniformly scaling up the depth, width, and resolution while scaling down the model instead of arbitrarily scaling these factors, as observed in conventional practice^36^. For example, the ResNet architecture is scaled up by adding more layers to improve accuracy. However, this approach results in increased computational complexity and vanishing gradient problems. EfficientNet addresses this issue by exploring the relationship between increases in each dimension using a compound coefficient. In addition, unlike other CNN models, it uses a new activation function called Swish instead of a Rectifier Linear Unit (ReLU). EfficientNetV2 is an improved version of EfficientNet with better training speed and parameter efficiency^37^. The EfficientNetV2 model employs a neural architecture search (NAS) to optimize model accuracy, size, and training speed. In this case, the EfficientNetV2-B0 model was used as the network, and fine-tuning was performed using a pretrained model with the Imagenet21k dataset. Fine-tuning uses the weights of the trained model and can thus achieve high accuracy with a small number of training images. The number of epochs was set to 50, and the batch size was set to 32 for training. Adam was used as the optimizer, and the dropout was set to 0.3. In this study, early stopping was also employed to prevent overfitting and automatic termination was performed when the validation loss did not improve by more than 0.001 for five consecutive epochs. These analyses were performed using the NVIDIA DGX Station A100. The environment was as follows: OS: Ubuntu 18.04.4, GPU: Tesla V100 32 GB, CUDA Version: 11.2, CPU: Intel Xeon CPU E5-2698 v4. In addition, we used the version 1.2.2 of Keras-efficientnet-v2^38^ on the TensorFlow backend.

### Evaluation metrics

The overall accuracy, Cohen’s Kappa coefficient, and macro average F1 score were used for evaluation. Although the overall accuracy is widely used in assessments, proper evaluations become difficult in cases of imbalanced data. Therefore, we also used Cohen’s Kappa coefficient and the macro average F1 score, which can be used to assess unbalanced data. For evaluation, the following equations were used. Regarding the formula of Cohen’s Kappa, ${\text{P}}_{o}$ is the observed agreement between ground truth and prediction. ${\text{P}}_{e}$ is the hypothetical chance of the ground truth and predictions arriving at the same number. The models were evaluated at different periods: morning, afternoon, and evening. For example, test images taken in the morning were used to assess the models trained based on the morning images; in addition, mixed models that contained images from all periods (morning, afternoon, and evening) were trained and tested.

$${\text{Accuracy}} = \frac{{\left( {{\text{TP}} + {\text{TN}}} \right)}}{{\left( {{\text{TP}} + {\text{FP}} + {\text{TN}} + {\text{FN}}} \right)}}\quad {\text{Kappa}}(k) = \frac{{{\text{P}}_{o} - {\text{P}}_{e} }}{{1 - {\text{P}}_{e} }}\quad {\text{Macro}}\,{\text{F}}1\,{\text{score}} = \frac{{\sum\nolimits_{i = 1}^{n} {{\text{F1}}\,{\text{score}}_{i} } }}{{{\text{Precision}} + {\text{Recall}}}}$$

## Results

### Morning model

**Figure 3:**
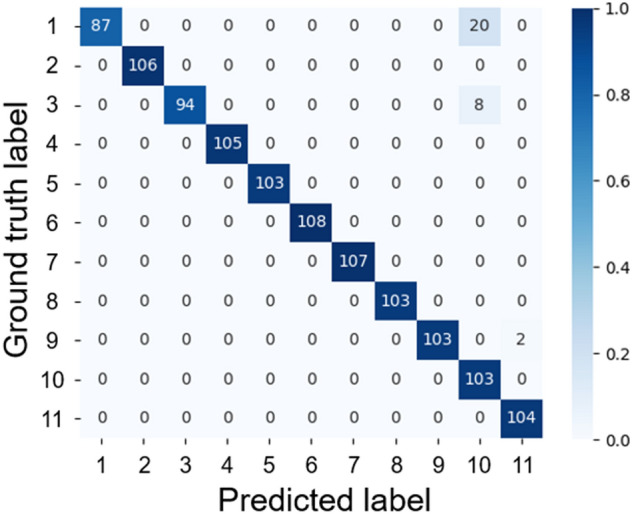
Confusion matrix of morning model. The vertical axis shows the ground truth, and the horizontal axis shows the model’s prediction results. The numbers on the axes indicate the individual numbers of *A. japonicus*; the number in each cell indicates the number of classified images; and the color of each cell indicates the percentage of images in each class.

The results of the morning model are shown in Fig. 3 and Table 2. These results are also shown in Supplementary Table 2. All individuals were correctly identified with an accuracy of 97.04%, a Kappa coefficient of 0.97, and an F1 score of 0.98. The identification results for each individual are presented in a confusion matrix. The vertical axis represents the ground truth, the horizontal axis represents the class predicted by the model, and each number represents an individual number. The number in each cell represents the number of identified images, and the color of each cell indicates the percentage of images per ground truth. For example, light blue indicates a ratio of 0.0, indicating that no image was classified as that cell. In contrast, dark blue indicates a ratio of 1.0, meaning that all ground truth images were classified to that cell. The morning model misclassified individual No. 1 as No. 10 in 20/107 (18.69%) images and individual No. 3 as No. 10 in 8/102 (7.84%) images. In addition, individual No. 9 images were misclassified as No. 11 by 2/105 (1.90%) images.

**Table 2:** Identification results and comparison of each model.

	Overall accuracy	Kappa	F1 score
Morning model	97.04	0.97	0.98
Afternoon model	94.36	0.94	0.92
Evening model	86.86	0.85	0.98
Mixed model	99.86	0.99	0.99

### Afternoon model

**Figure 4:**
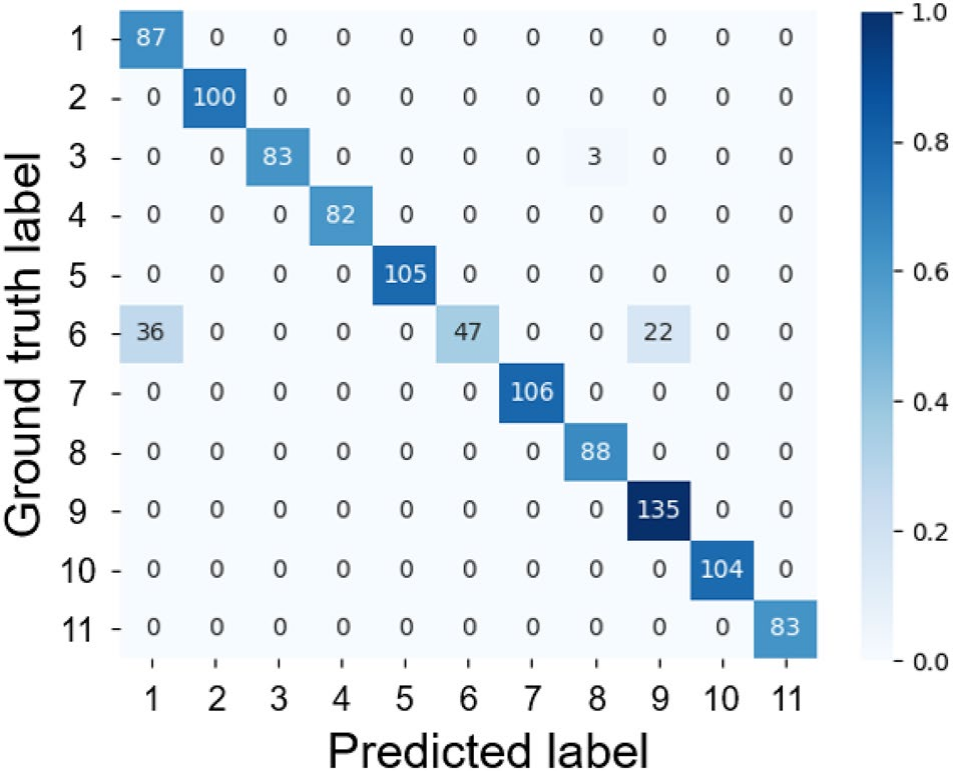
Confusion matrix of the afternoon model.

The results of the afternoon model are shown in Fig. 4 and Table 2. All individuals were correctly identified with an accuracy of 94.36%, a Kappa coefficient of 0.94, and an F1 score of 0.92. The model misclassified 58/105 images (55.24%) of the individual No. 6, 36/105 images as No. 1, and 22/105 images as No. 9 (Fig. 4). In addition, individual No. 3 were misclassified as No. 8 by 3/86 images (3.49%).

### Evening model

**Figure 5:**
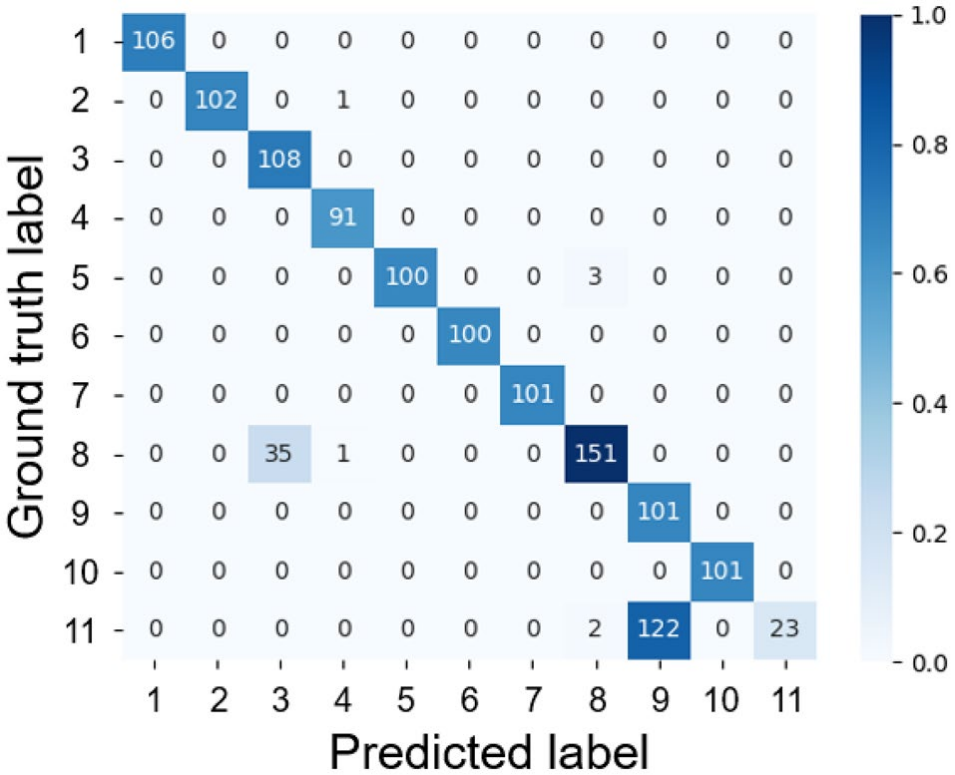
Confusion matrix of the evening model.

**Figure 6:**
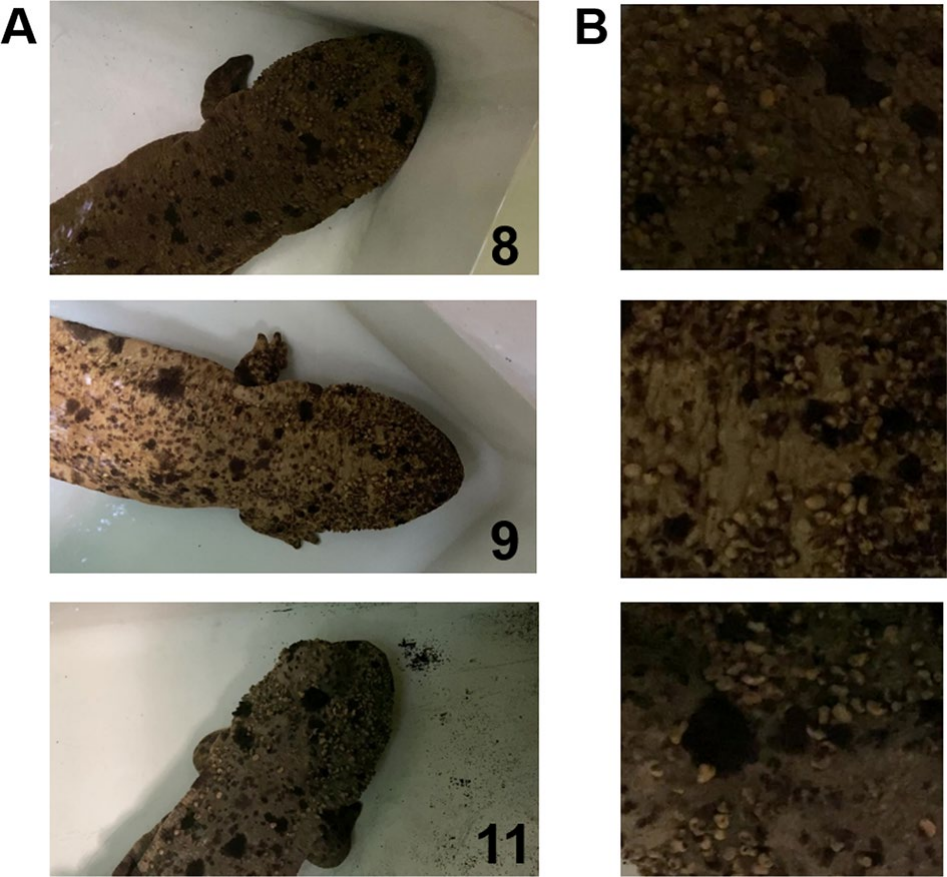
Misclassifications of the evening model, including 124/147 images (84.35%) of individual No. 11, 122/147 images (82.99%) as individual No. 9, and 2/147 images (1.36%) as individual No. 8. A shows the spots for each individual, and B shows the test image of each individual.

The results of the evening model are shown in Fig. 5 and Table 2. Compared to the morning and afternoon models, the accuracy was lower, with an accuracy of 86.86%, Kappa coefficient of 0.85, and F1 score of 0.98. The evening model misclassified 124/147 images (84.35%) of individual No. 11 (Fig. 6), 122/147 images (82.99%) as No. 9, and 2/147 images (1.36%) as No. 8. In addition, 36/187 images (19.25%) of No. 8 individuals were misclassified, 35/187 images as No. 3, and 1/187 images as No. 4. Futhermore, 3/103 images (2.91%) of individual No. 5 were misidentified as No. 8 and 1/103 images (0.97%) of individual No. 2 were misclassified as No. 4.

### Mixed model

**Figure 7:**
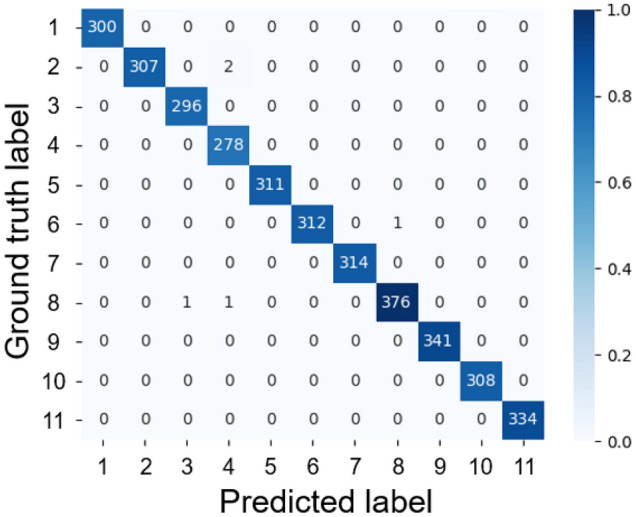
Confusion matrix of the mixed model.

The mixed-model results are shown in Fig. 7 and Table 2. The accuracy was 99.86%, Kappa coefficient was 0.99, and F1 score was 0.99. Although there were some misidentifications in the images of individuals No. 2, No. 6, and No. 8, the model correctly identified almost all individuals.

## Discussion

This study applied deep learning approaches for the individual of endangered amphibians using deep learning. In most wildlife studies employ artificial tag attachments to identify individuals by capturing animals^14^. However, physical tags have several issues, such as the stress associated with capture and tag attachment and the impact of the tags itself. In contrast, non-invasive methods, such as photoidentification, have a lower impact on animals, although they also present certain disadvantages. For example, photographic matching requires identification skills and cannot be applied to large datasets because of its labor-intensive nature and human errors. Although deep learning can overcome these challenges, it has only been applied to a few taxa, which mainly include mammals. Thus, research on amphibians using deep learning is lacking despite its importance for conservation. Our study demonstrated the effectiveness of a new identification method using deep learning for one of the world’s largest amphibians that is currently threatened with extinction. We found that the head spot pattern was suitable for individual identification, and an accuracy of 99.86% was achieved by applying EfficientNetV2 to smartphone images without conventional feature extraction. The high performance obtained with smartphone images suggests that combining this method with citizen science could contribute to amphibian conservation. Moreover, our approach could be applied to other amphibian species at a low cost.

The accuracy of the mixed model was 99.86%, the Kappa coefficient was 0.99, and the F1 score was 0.99. Previous studies that used deep learning for individual identification achieved accuracies of 92.5% for chimpanzees^20^, 96.3% for pandas^18^, and 83.9% for brown bears^19^. One reason for the high accuracy in this study was the high similarity between the training and test images. Whereas there was significant variation in the date and location of training images in the previous studies, the training and test images used in this study were obtained over only two days. Furthermore, the high accuracy was probably due to the low variation in the images because the *A.japonicus* did not move much during shooting and the clear images obtained for the spots in water in a captive environment. When photographing *A.japonicus* on land, individuals move relatively fast and their body surfaces reflect light. These problems can be mitigated in water. Because markings for individual identification can be recorded under natural conditions when reflection from the water surface is suppressed, photographing underwater individuals is recommended for aquatic amphibians. Another factor contributing to the high accuracy was that the images contained only the spots used for analysis. In fact, the accuracy was reduced when images were used without cropping, especially in the evening model (Supplementary Table 3).

The performance of the evening model was lower than that of the morning and afternoon models, which may be due to the quality of the images resulting from the light conditions. In particular, individual No. 11 in the evening model was misclassified in 124 out of 147 (84.35%) images. These images could not be classified correctly because the spots were not clearly photographed (Fig. 6). In addition, confusion may occur because the features of different individuals extracted by AI are similar. Furthermore, the fact that only the head was used for analysis may have contributed to the limited information in the images. In the case of the expert, observers identify individuals based on the characteristic pattern of spots on the whole body, not just the head, to isolate slight differences between individuals. Therefore, it is necessary to verify how the performance varies depending on the areas for individual identification. The selection of the measurement region for identification is important and has a significant effect on the accuracy of the model. For example, in the case of seals, the accuracy was 59% for fur-based identification^39^ but improved to 88% for face-based identification^17^. Arzoumanian et al*.*^40^ achieved more than 90% pair image matching using flank spot patterns for the identification of whale sharks but reported that image matching failed when photographs were obtained at oblique angles of more than 30°.

The mixed model exhibited the best results. One possible reason is the quality of the training images. Since training images of the morning and afternoon models clearly photographed spots in relatively luminous environments, the image brightness could have contributed to the performance. Although the light intensity in our study was not measured, the solar radiation (MJ/m^2^) recorded at the Hiroshima Local Meteorological Observatory was high in the morning and afternoon when the images were captured. Previous studies also indicated that the accuracy of AI models is affected by light intensity and shadow in training images^21^. Therefore, it is important to obtain images in the appropriate environment, depending on the research purpose. As the second reason, the size of the training data in the mixed model could also have increased the accuracy. Tabak et al.^41^ also indicated that the ability of the model to recognize species increased with the size of the training dataset for the species. Although it is challenging to collect training images of animals with small populations, such as endangered species, cooperation with zoos and aquariums will enable the efficient creation of training datasets. Thus, collaboration among ecologists, information scientists, and zoo curators will be required in the future.

This study demonstrated the benefits of using deep learning to identify individuals; however, certain limitations should be noted. First, we used individuals in captivity, which facilitated uniform shooting conditions. In situations where images are captured in the wild, the background, light conditions, water flow, and direction of the target species differ, which leads to high image variation, thereby affecting the identification accuracy. Prior studies have shown that the accuracy is lower when individual identification is conducted in the field than in captivity, with 92% accuracy reported for chimpanzees in captivity and 77% accuracy in the wild^42^. In the future, individual identification using the proposed method should be verified for practical application in the field. Second, the data were obtained over two days; therefore, the verification of this method for long-term individual identification is needed. In particular, long-term monitoring is important for surveying long-lived target species and for their conservation. Thirdly, relatively large adult *A.japonicus* with a total length of over 50 cm generally show little change in their spots over time. However, few studies have examined changes in the spot patterns of *A.japonicus* over their lifetime, although the study of Tochimoto^32^ demonstrated continuous identification from the body pattern over eight years. For other species, regions that do not change over long periods should be used for individual identification. For example, Bauwens et al*.*^43^ analyzed images of over 900 European adders (*Vipera berus*) over 12 years and found that head scale patterns did not change and were useful markers. Therefore, future studies are needed to determine the effects of aging and weight changes on the spot visibility of *A.japonicus*. In addition, our method uses pre-captured images to identify individuals. Therefore, identifying new individuals not included in the training images is challenging. This means that applying our predictive model to other *A.japonicus* will be difficult because our training datasets contained only 11 individuals. In the future, more individuals must be annotated to utilize our model for field surveys. Finally, the ability to identify unknown individuals was not tested in this study. However, it can be tested by the following procedure. If there are images from five individuals (e.g., A to E), an AI model can be created without including the images of one individual (e.g., E). When E’s image is tested using this model, if it is classified as a particular individual (e.g., A) with high probability, the model may not be able to sufficiently identify unknown individuals. On the other hand, if the probabilities of E’s classification as known individuals (A to D) are uniformly low, this indicates that the model correctly identified the unknown individual. Applying the same process to other individuals will enable evaluation of the ability of the model to identify unknown individuals.

The approach implemented in this study, which combines images and deep learning, can identify target species inexpensively without the need for capture. Moreover, although conventional machine learning requires the design of species-specific algorithms for feature extraction, which are challenging to implement^16,44^, highly accurate individual identification was achieved in our study using images alone. This simplicity is a major advantage, and we believe that similar methods can be applied to other species of amphibians. The findings of this study can contribute to the conservation of amphibians, which are threatened with extinction worldwide. Furthermore, our technique enables applications such as estimating population size. For example, the proportion of marked individuals is needed when applying the mark-recapture method. Previously, target species were marked by attaching tags (e.g., PIT tags) to distinguish between new and re-captured individuals. Our method suggested the possibility of using photographs as an alternative to tags. In addition, our research demonstrated the feasibility of individual identification using smartphone images. Smartphones are widely available at a low cost; therefore, applying this methodology will significantly advance conservation via combination with citizen science. For example, whale shark research has accumulated over 43,000 images with the help of 3400 researchers and citizen scientists, and over 3800 individuals have been identified^45^. Such a large database can prevent the illegal release of individuals and trade in wildlife^46^. For example, *A. japonicus* can go away on land from the streams after heavy rain, and sightings of such animals have attracted attention on social networking sites, such as Twitter. In this case, individual identification from the images may assist in determining and releasing the original habitat. In addition, our technique could be applied to detect invasive species. Recently, hybridization between *A. japonicus* and non-native *A. cf. davidianus* has become a serious issue. Although capturing hybrids in the wild is necessary, they have a spot pattern that inherits the characteristics of both native and non-native species if they are F1 offspring. Therefore, suspected hybrids are identified by visual screening and DNA analysis. However, the visual screening of hybrids is challenging for the public because it requires identification skills. In the future, if hybrid species could be identified from the images, our approach would be a useful tool for the rapid assessment of hybrid species by citizens. Additionally, the identification of individuals may be possible after their illegal capture. For example, a case of human transportation of an individuals from Hyogo Prefecture to Shiga Prefecture (a distance of over 100 km) in 2022 was revealed by its PIT tag. The purpose of its transportation is unknown; however, the unauthorized capture and movement of this species are prohibited by law. A citizen science survey can prevent the illegal trade and release of *A. japonicus* by constructing a database that allows the matching of individuals. Moreover, ecological information such as age at maturity and migration patterns can be obtained by collecting images over a long period. Understanding the life history is essential for conservation, particularly for long-lived species. Combining images with deep learning enables inexpensive long-term monitoring and offers new opportunities to contribute to ecology.

## Conclusion

In this study, we applied deep learning to identify the endangered amphibian, *A. japonicus*. Our study revealed that deep learning-based individual identification, which has previously focused on mammals, is feasible for amphibians and that high performance can be achieved using smartphone images. Individuals were identified with high accuracy by clearly photographing the head spots while suppressing reflection from the water surface. This method enable the stress-free capture of images of the target species in natural conditions without interfering with their movements. Image-based individual identification is non-invasive and inexpensive, and large datasets can be automatically processed when combined with deep learning. In addition, owing to the widespread popularity of smartphones, this method can contribute to the conservation of species with natural markings.

### Supplementary Information

Supplementary Information.

## Data Availability

The source code for running EfficientNetV2 is available from https://github.com/kosuke-takaya/efficientnet_v2.git. In addition, other data supporting this study’s findings are included within the article and supplementary files.
