# Supplementary material for: Individual identification of endangered amphibians using deep learning and smartphone images: case study of the Japanese giant salamander (Andrias japonicus)
Source: Sci Rep. 2023 Sep 27;13:16212. doi: 10.1038/s41598-023-40814-1 (PMC10533547; doi:10.1038/s41598-023-40814-1)

**Individual identification of endangered amphibians using deep learning and smartphone images: case study of the Japanese giant salamander (*Andrias japonicus*)**

Kosuke Takaya*, Yuki Taguchi and Takeshi Ise

*Corresponding author

**Supplementary information**


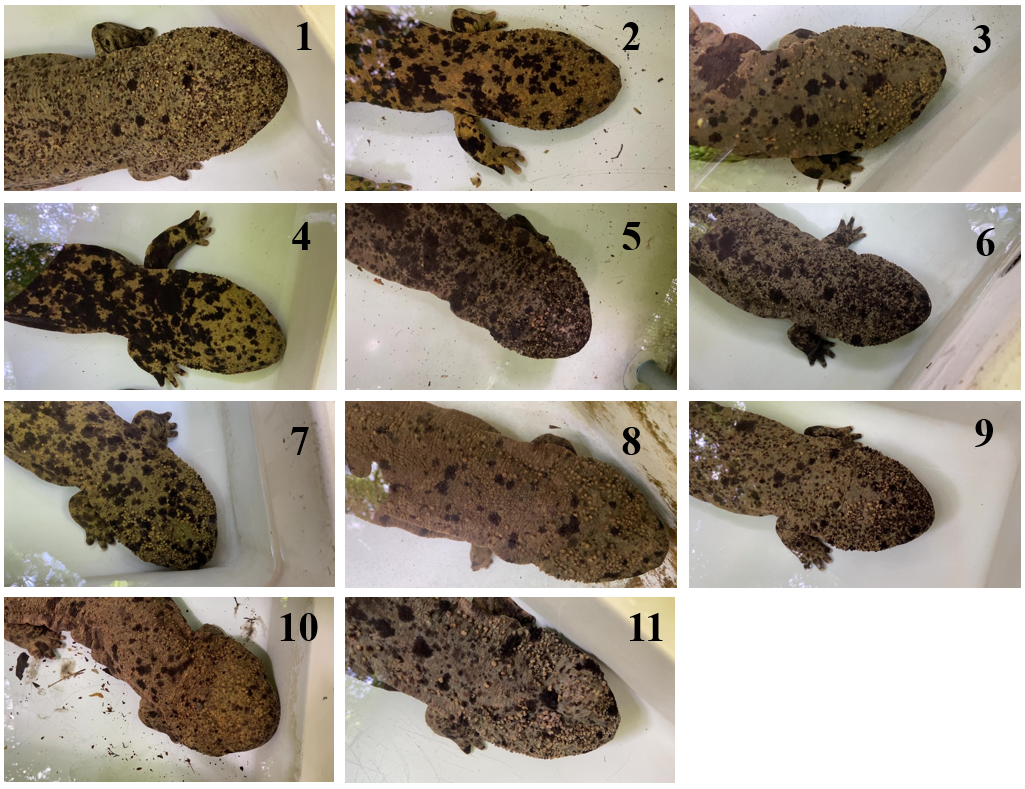


Supplementary Fig. 1. Eleven individuals were used in this study.


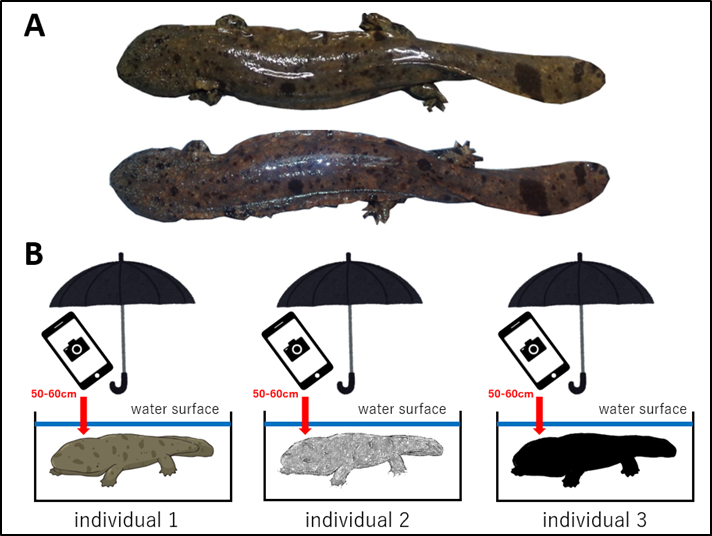


Supplementary Fig. 2. Methods of photographing salamanders. A: Body surface reflections differed greatly between images when photographed on land, even for the same individual; B: Photographs of underwater individuals were taken from under the umbrella to reduce reflections on the water's surface. An iPhone11 equipped with a 12-megapixel camera was used for the photo shoot.


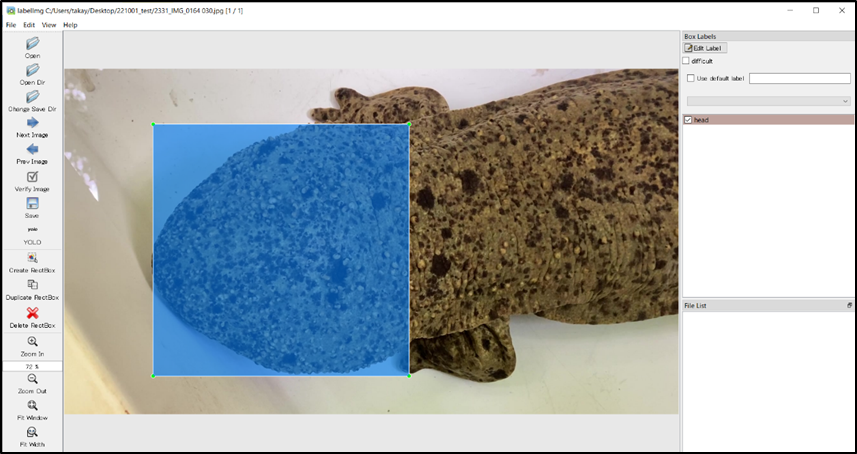


Supplementary Fig. 3. Annotation data preparation using the LabelImg annotation tool. The blue rectangle indicates the labeled regions. To detect only the head, the rostrum and head width were labeled.

Supplemental table 1. The number of training and test images selected for each individual. Training images were obtained on August 20, and test images on August 21.

Supplemental table 2. Identification results by each AI model.

Supplementary Table 3. Performance comparison of models created with 60% cropped and head image models. Except for the afternoon model, the performance of the model created with the 60% cropped image was better.


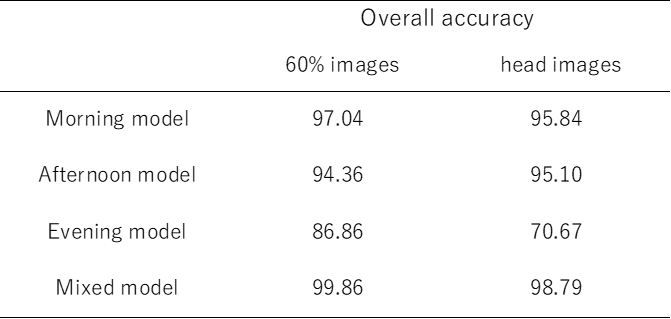

Supplement: Supplementary file 1 — Supplementary Information. [file 41598_2023_40814_MOESM1_ESM.docx]
